# Effects of Whey Protein or Its Hydrolysate Supplements Combined with an Energy-Restricted Diet on Weight Loss: A Randomized Controlled Trial in Older Women

**DOI:** 10.3390/nu14214540

**Published:** 2022-10-28

**Authors:** Yue Sun, Chenjie Ling, Linsheng Liu, Jianwei Zhang, Jian Wang, Xing Tong, Khemayanto Hidayat, Mengting Chen, Xiaofang Chen, Hui Zhou, Jiaying Xu, Liqiang Qin, Wanzhan Zhu, Jing Yang

**Affiliations:** 1 Department of Nutrition and Food Hygiene, School of Public Health, Soochow University, Suzhou 215000, China; 2 Department of Clinical Nutrition, Dushu Lake Hospital Affiliated to Soochow University, Suzhou 215000, China; 3 Department of Pharmacy, The First Affiliated Hospital of Soochow University, Suzhou 215000, China; 4 Department of Clinical Nutrition, Suzhou Municipal Hospital, Suzhou 215000, China; 5 School of Environment and Civil Engineering, Jiangnan University, Wuxi 214000, China; 6 Laboratory Center, Medical College of Soochow University, Suzhou 215000, China; 7 Department of Endocrinology and Metabolism, The First Affiliated Hospital of Soochow University, Suzhou 215000, China; 8 Suzhou Industrial Park Centers for Disease Control and Prevention, Suzhou 215000, China; 9 State Key Laboratory of Radiation Medicine and Protection, School of Radiation Medicine and Protection, Soochow University, Suzhou 215000, China; 10 Department of Clinical Nutrition, The First Affiliated Hospital of Soochow University, Suzhou 215000, China

**Keywords:** whey protein, whey protein hydrolysate, body composition, gut microbiota, serum metabolome

## Abstract

An energy-restricted weight-loss approach has limitations when it used in the elderly, especially because of muscle loss. We aimed to assess the effects of whey protein (WP) or WP hydrolysate (WPH) combined with an energy-restricted diet (ERD) on weight reduction and muscle preservation in older women with overweight and obesity. A total of 60 women were randomized to the control (ERD), WP (ERD + 20 g/d WP) or WPH (ERD + 20 g/d WPH) group, using a 1:1:1 allocation ratio. After an 8-week intervention, body composition, gut microbiota, and serum metabolomics changes were compared among the three groups. The reductions in body weight (−1.11 ± 1.11 vs. −2.34 ± 1.35, *p* < 0.05), BMI (−0.46 ± 0.45 vs. −0.97 ± 0.54, *p* < 0.05), and body fat (−0.70 ± 0.92 vs. −2.45 ± 1.65, *p* < 0.01) were higher in the WPH group than in the control group. Body fat (%) was significantly decreased in the two protein groups. Fat-free mass did not significantly change among the three groups. Serum metabolomics showed that the tricarboxylic acid cycle pathway was upregulated in the WPH group. No significant changes in microbiota were observed among the groups. In conclusion, WP or WPH supplementation combined with an energy-restricted diet benefits older women during weight loss. WPH was more effective, possibly due to increased energy metabolism.

## 1. Introduction

Obesity has become a major global public health issue. Overweight and obesity have increased rapidly in the past four decades in China, and the latest national prevalence rate was 34.3% for overweight and 16.4% for obesity in adults [1]. Given the current increase in life expectancy, the prevalence of obesity has also increased steadily among older adults. Obesity increases the risk of many diseases, including diabetes, cardiovascular disease, and some cancers.

Weight loss is important for older adults with obesity, but a conventionally adopted low-calorie diet is likely to exacerbate the age-related loss of skeletal muscle (sarcopenia). Unfortunately, the mortality risks of muscle loss may outweigh the potential benefits of weight loss in the elderly [2]. Resistance training is helpful for muscle preservation. Thus, most weight-loss trials have used resistance training combined with diet control [3,4]. However, it is difficult for the elderly to implement and adhere to the resistance training program. Therefore, investigating whether dietary adjustment alone could achieve the goal of reducing fat and preserving muscle is important.

Intervention studies in Western countries have shown that high protein intake attenuates muscle mass loss in the natural progress of aging [5] and during energy restriction [6,7]. Whey protein (WP) is one of the highest-quality proteins and is shown to stimulate muscle protein synthesis effectively [8]. There are different types of WP, including concentrate, isolate, hydrolysate, and native whey protein. Based on a survey of Korean university students, whey protein hydrolysate (WPH) was the most common type of protein supplement [9]. Because WPH is digested and absorbed more rapidly than intact WP [8], its effects are worth observing and comparing in one trial.

Emerging evidence showed that gut microbiota is an important factor linked to obesity by altering host energy balance [10]. Diet can influence gut microbiota composition [11]. Previous clinical studies on gut microbiota have focused on indigestible carbohydrates, whereas the effects of dietary proteins on the gut microbiota have been much less studied [12]. Meanwhile, metabolites are also attractive biomarkers for understanding perturbations in response to dietary interventions [13,14]. It has been reported that high serum BCAA is related to obesity and insulin resistance [15]. Thus, it would be interesting to investigate whether BCAA-enriched WP and WPH regulate serum BCAA concentrations and other metabolites. We designed the present intervention study to assess the effects of an energy-restricted diet combined with WP or WPH in older women with overweight and obesity on weight loss and muscle preservation.

## 2. Methods

### 2.1. Participants

Older women aged from fifty to eighty years with overweight and obesity (body mass index [BMI]: 24–35 kg/m^2^) were recruited from local communities via advertisement and word-of-mouth from September 2019 to October 2020. Exclusion criteria included: (1) those with digestive diseases, such as gastrointestinal ulcers, inflammatory bowel disease, pancreatitis, severe vomiting, diarrhea, intestinal obstruction, or other conditions that affect gastrointestinal digestion and absorption; (2) those with severe cardiovascular disease, such as heart failure, uncontrolled blood pressure (systolic blood pressure, SBP  >  160 or diastolic blood pressure, DBP > 100 mmHg), myocardial infarction, and severe arrhythmias; (3) those with severe pulmonary disease, major liver dysfunction, cognitive impairment, or uncontrolled blood glucose that could adversely influence the results; (4) those with renal insufficiency (the serum creatinine level exceeds the upper limit of normal); (5) those who were unwilling to eat the prescribed diets, disagreed with maintaining their activity level throughout the study, recently on weight loss, or using supplements such as protein powder, fish oil, antioxidants, and anti-inflammatory products.

### 2.2. Study Design

**Figure 1:**
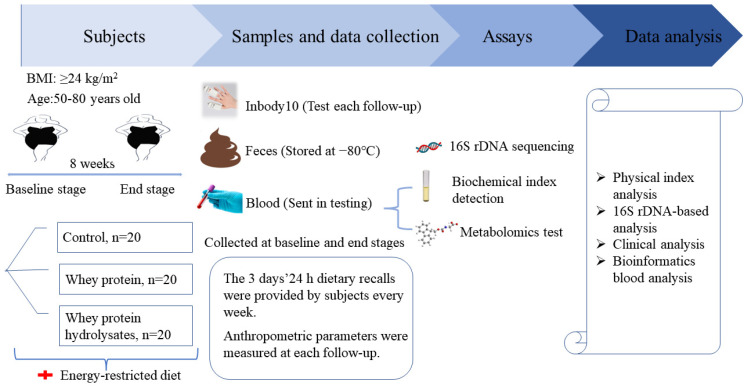
Experimental design.

The study was a randomized, single-blinded, controlled eight-week weight-loss intervention trial (Figure 1). Participants were blinded to the intervention they performed. All participants were given individual instructions by a dietitian to consume an energy-restricted diet during the intervention. An energy-restricted diet is a reduction of 500 kcal from the target energy calculated based on a person’s ideal weight. The diet provided approximately 55%, 25%, and 20% energy from carbohydrates, fat, and protein, respectively [16]. The participants were instructed on the type and amount of food consumed in three daily meals. Examples of recipes and methods for replacing the same food were provided.

Then, the participants were randomly assigned to the control, WP, and WPH groups. The randomization sequence was created by computer software. The research staff opened the sequentially numbered opaque envelope that contained the product assignment. An individual unassociated with the study prepared the envelopes. WP (Arla, Inc., Viby J, Denmark, containing 7.6 g protein/10 g) and WPH (Arla, Inc., Viby J, Denmark, containing 8.4 g hydrolysate protein/10 g) were packaged in 10 g per sachets (Table S1). All the sachets had an identical appearance. The participants were instructed to dissolve one sachet of protein powder in approximately 250 Ml of water and consume the drinks together at breakfast and dinner. Thus, 20 g supplements were ingested per day by the participants in the WP and WPH groups. No additional intervention was provided in the control group.

At baseline, energy-restricted diet and intervention conduction, participants were asked to record their food consumption (two weekdays and one weekend day). Macronutrient content of the diet was assessed using Golden Key Nutrition Expert System (Shanghai Ying Kang Computer Technology Co., Ltd., Shanghai, China). Daily physical activity was evaluated using the physical activity scale for the elderly (PASE) [17]. Professional personnel assisted each participant in controlling their calories and intervention through personal visits and WeChat twice (during the first 4 weeks) or once (during the last 4 weeks) a week. The contents included the execution of a diet plan and/or protein supplements, and discomfort during the study. At baseline and the end of the intervention, measurements of the body composition, blood measurements, metabolomics, and gut microbiome were performed.

The trial was approved by the Ethics Committee of Soochow Universityand was registered at the Chinese Clinical Trial Registry with the registration number ChiCTR-IPR-2200057152. Written informed consent was obtained from each participant.

### 2.3. Body Composition

Participants were required to wear light clothing and remove all metal objects from their bodies while undergoing this test. Body composition, including body fat, visceral fat, fat-free mass, and muscle mass, was measured by inBody S10 (Biospace Co., Ltd., Seoul, Korea).

### 2.4. Blood Pressure Measurement

The participants were asked to sit quietly for at least 15 min, and blood pressure (BP) was measured three times at 5 min intervals using an automatic BP measuring device (Shenzhen Jiakang Technology Co., Ltd., Shenzhen, China). Additional measurements were taken when BP values varied by ≥10 mmHg.

### 2.5. Analysis of Glucose, Lipid Metabolism, and Inflammatory Factors

For the biochemical analysis, venous blood samples were collected after overnight fasting of at least 10 h. The serum was separated through centrifugation and stored at −80 °C. Fasting blood glucose (FBG), triglycerides (TG), total cholesterol (TC), low-density lipoprotein cholesterol (LDL-C), high-density lipoprotein cholesterol (HDL-C), and uric acid (UA) were measured using a Hitachi Automatic Biochemical Analyzer 7180 (Hitachi, Ltd., Tokoy, Japan. Fasting insulin (FINS) was measured using a fully automatic electrochemiluminescence analyzer Roche Cobas E602 (Roche Diagnostics, Mannheim, Germany). Insulin sensitivity was assessed by homeostasis model assessment–insulin resistance (HOMA-IR), which was calculated as follows: HOMA-IR = FBG (mmol/L) × FINS (μU/mL)/22.5.

Circulating markers of inflammation include C-reactive protein concentration (CRP), tumor necrosis factor α (TNF-α), and free fatty acids (FFA). CRP was determined using an automatic electrochemiluminescence analyzer Roche Cobas C702 (Roche Diagnostics, Mannheim, Germany). TNF-α and FFA were determined using the multifunctional enzyme marker Tecan sunrise (Tecan, Mannedorf, Switzerland).

### 2.6. Nontargeted Metabolomics

Metabolomics were determined using liquid chromatography–mass spectrometry (LC-MS, Thermo Fisher Scientific, Waltham, MA, USA). First, 50 μL serum was added to 200 μL mixed solvent (methanol: acetonitrile = 5:3, *v*/*v*) and shaken for 3 min to precipitate protein. After centrifugation at 18,000 rpm at 4 °C for 10 min, 120 μL supernatant was absorbed and transferred to the vial for sample injection. Another 20 μL of each sample was taken, and the mixed samples were used as QC samples after processing according to the above method. Before the analysis, three consecutive samples were injected to ensure the good stability of the instrument. QC samples were added between every 10 samples during the analysis for data correction.

LC-MS analysis conditions: Chromatographic conditions: Waters HSS T3 column (2.1 × 100 mm, 1.8μm). The mobile phase consisted of 0.1% formic acid water (A)-acetonitrile (B), and the volume flow rate was 0.3 mL/min. The column temperature was 40 °C. The injection volume was 2 μL. Ms conditions: ionization mode was electrospray (ESI) positive ion mode and negative ion mode, the ion source voltage was 3500 V (positive ion) and −2500 V (negative ion), capillary temp was 325 °C, and aux gas heater temp was 300 °C. The scanning range of primary mass spectrometry parent ion was 100–1500 *m/z* with a resolution of 70,000, and the scanning range of secondary mass spectrometry (N)CE was 30 with a resolution of 17,500.

### 2.7. Stool Sample Collection and DNA Extraction

Stool samples were collected at baseline and end of the intervention. After normal defecation, a small amount of fresh feces was collected from a toilet paper with a swab. The cotton swabs stained with feces were immersed in the liquid of the sampling tube and stirred to elute the feces into the preservation liquid of the sampling tube. Stool collection was completed by the subjects according to the standard collection method. The researcher stored the feces samples in the −80 °C refrigerator. Afterward, DNA was extracted using a sodium dodecyl sulfonate (SDS) lysate freezing–thawing method. Total bacterial genomic DNA was extracted using the GHFDE100 DNA isolation kit (Hangzhou Guhe Information Technology Co. LTD, Hangzhou, China) according to the manufacturers’ instructions. The quantity and quality of the extracted DNA were measured using a NanoDrop ND-1000 spectrophotometer (Thermo Fisher Scientific, Waltham, MA, USA) and agarose gel electrophoresis, respectively. The above process was completed by the Hangzhou Guhe Information Technology Co., Ltd. (Hangzhou China)

### 2.8. Gut Microbiota Analysis

The 16S rRNA sequencing was performed by Hangzhou Guhe Information Technology Co., Ltd. The polymerase chain reaction (PCR) was performed as described [18]. The PCR products were purified using AMPure XP Beads (Beckman Coulter Inc., Brea CA, USA) and quantified using a PicoGreen dsDNA Assay Kit (Thermo Fisher Scientific, Waltham, MA, USA). Then, sequencing was performed on an Illumina Novaseq6000 pair-end 2 × 150 bp platform. Clean data were obtained after data dividing, merging, and filtering. The operational taxonomic units (OTUs) were clustered at 97% similarity using the Vsearch software. According to the representative sequences of OTUs, species annotation based on the SILVA database was performed using VSEARCH. Then, further analysis could be conducted.

### 2.9. Statistical Analysis

The sample size of the trial was estimated using PASS software based on a previous study from our team. Subjects who completed the study were included in the data analysis. The statistical analysis software used in the research was open source R4.02 software and SPSS26.0 (IBM Corp., Armonk, NY, USA). The graphs were drawn by GraphPad prism 8. Data were expressed as mean ± SD (normal distribution) or median (interquartile range) (abnormal distribution). Comparison among the three groups was performed by one-way ANOVA or Kruskal–Wallis H test. Further, comparisons between groups were performed using LSD or the Mann–Whitney U test. Paired t-test or Wilcoxon signed-rank test were used to compare differences before and after the intervention. One-way ANOVA and LSD were used to compare the α diversity among the three groups, and paired t-test was used for comparison before and after the intervention. Kruskal–Wallis H test and the Mann–Whitney U test were used to compare the gut microbiota composition among the three groups, and Wilcoxon signed-rank test was used for comparison before and after the intervention. LDA Effect Size (LEfSe) analysis (LDA > 2) was performed on the website (http://huttenhower.sph.harvard.edu/galaxy/, accessed on 20 March 2022.).

Analysis of metabolomics: Using compound discovery software to preprocess the original data, such as peak identification, alignment, noise filtering, peak area normalization, and compound identification, a data matrix containing metabolite retention time, mass charge ratio, and intensity was obtained. The results were imported into SIMCA-P 13.0 software for principal component analysis (PCA) and orthogonal partial least squares–discriminant analysis (OPLS-DA). Variables that meet the projected value of variable importance (VIP) > 1.0 and FDR < 0.05 were considered different variables. The different variables were entered into the MetaboAnalyst 5.0 (http://www.metaboanalyst.ca/, accessed on 15 October 2021) website for metabolic pathway analysis, and metabolic pathways with effect value greater than 0.08 were considered as potential target pathways.

## 3. Results

Sixty older women with overweight and obesity were enrolled. Twelve participants discontinued the study. Among them, seven withdrew for failure to maintain adequate compliance with the protocol, four were lost to follow-up, and one felt gastrointestinal discomfort. Finally, 48 women (18 in the control group, 16 in the WP group, and 14 in the WPH group) completed the study.

After randomization, no significant differences were noted in baseline body weight among the three groups. No significant differences were noted in body weight among those who completed or did not complete the study in the three groups. The baseline characteristics of the subjects who completed the study are shown in Table 1. The average age of the participants was 61.3 years old (SD = 7.73). The average body weight and BMI were 65.66 kg (SD = 5.15) and 27.21 kg/m^2^ (SD = 1.61), respectively. We collected the dietary intake and activity levels of the participants via dietary surveys and physical activity scales. The results showed that the average energy intake was 1458.17 kcal (SD = 301.23), with 13.3% from protein, 61.0% from carbohydrate, and 25.7% from fat. The average PASE was 112.98 (89.55, 133.18). 

After instructions by the dietitian, the average energy intake decreased by approximately 400–500 kcal. The average protein intake was 0.78 ± 0.05, 1.01 ± 0.07, and 1.08 ± 0.06 g/kg/d in the control, WP, and WPH groups, respectively. No significant differences in carbohydrate and fat intakes were observed among the three groups. Energy intakes were significantly higher in both WP (1126.80 (1027.80, 1187.55) vs. 1003.00 (919.75, 1088.50), *p <* 0.01) and WPH (1115.36 (1074.86, 1173.86) vs. 1003.00 (919.75, 1088.50), *p <* 0.01) groups than in the control group because of the additional protein supplements given in WP and WPH groups (Table 2).

Body weight, BMI, and body fat significantly decreased after intervention in the three groups. Comparisons among groups showed that significant differences were only found between the control and the WPH groups, with the latter achieving more decreases in body weight (−1.11 ± 1.11 vs. −2.34 ± 1.35, *p <* 0.05), BMI (−0.46 ± 0.45 vs. −0.97 ± 0.54, *p <* 0.05), and body fat (−0.70 ± 0.92 vs. −2.45 ± 1.65, *p <* 0.01). The percentage of body fat and the visceral fat area were significantly decreased in the WP and the WPH groups after the 8-week intervention. The WPH group had lower decreases in the percentage of body fat and visceral fat areacompared with the WP group, but the differences were not statistically significant. Fat-free mass did not differ statistically after the intervention in the three groups, although there was an increasing tendency for fat-free mass in the WPH group. (Table 3).

BP, metabolic, and inflammatory indices are shown in Table 4. No other significant differences in the changes of these indices were observed when compared between groups. In the WP group, serum TG was significantly decreased after the intervention. WP significantly increased FBG and FINS, resulting in an increased HOMA-IR. In the WPH group, SBP significantly decreased after the intervention. For the intercomparison of index changes, the HOMA-IR decrease was significantly greater in the WPH than in the WP group. 

**Figure 2:**
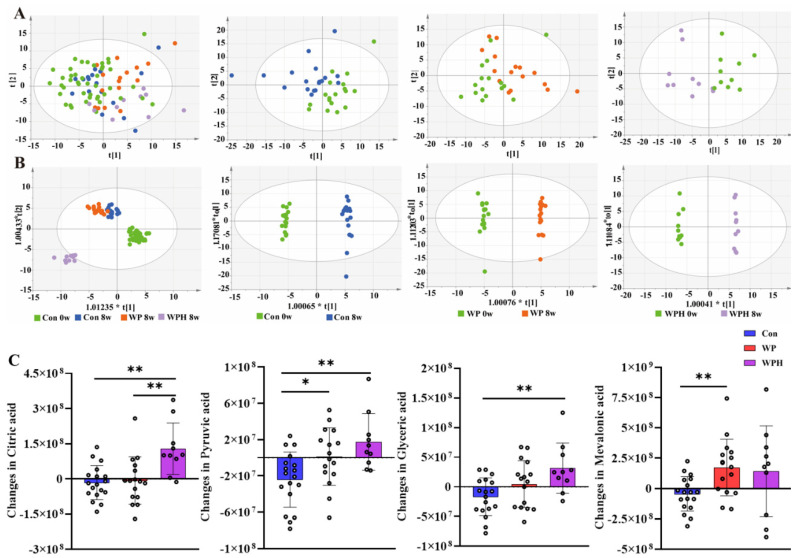
Serum metabolomics changed after the intervention. PCoA (**A**) and OPLS-DA score plots (**B**) comparing the serum metabolites before and after the intervention in each group. Changes in metabolic compounds in each group (**C**). * *p* < 0.05, ** *p* < 0.01.

Nine serum metabolites significantly changed in concentration after the intervention, and four metabolite changes had significant differences compared between groups (*p* < 0.05, FDR < 0.05) (Table S2). Among the four metabolites, mevalonic acid was significantly upregulated in the WP group compared with the control group. Citric acid, pyruvic acid, and glyceric acid were significantly upregulated in the WPH group compared with the control group. Citric acid was significantly upregulated in the WPH group compared with the WP group (Figure 2). The coordinated effects of change in metabolites might be enriched for pathways, and further analysis indicated that the TCA cycle pathways were upregulated in the WPH group.

**Figure 3:**
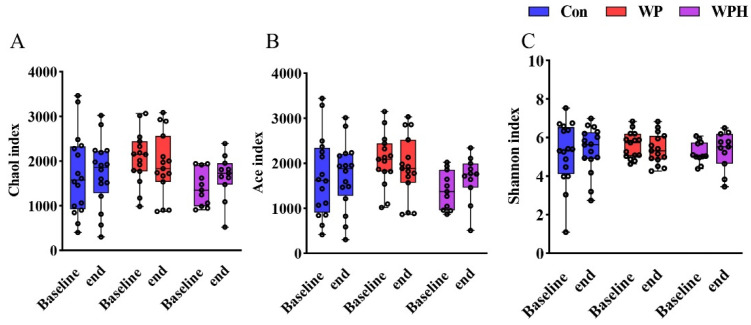
α diversity analysis is based on Chao1 (**A**), Ace (**B**), and Shannon (**C**) among groups.

**Figure 4:**
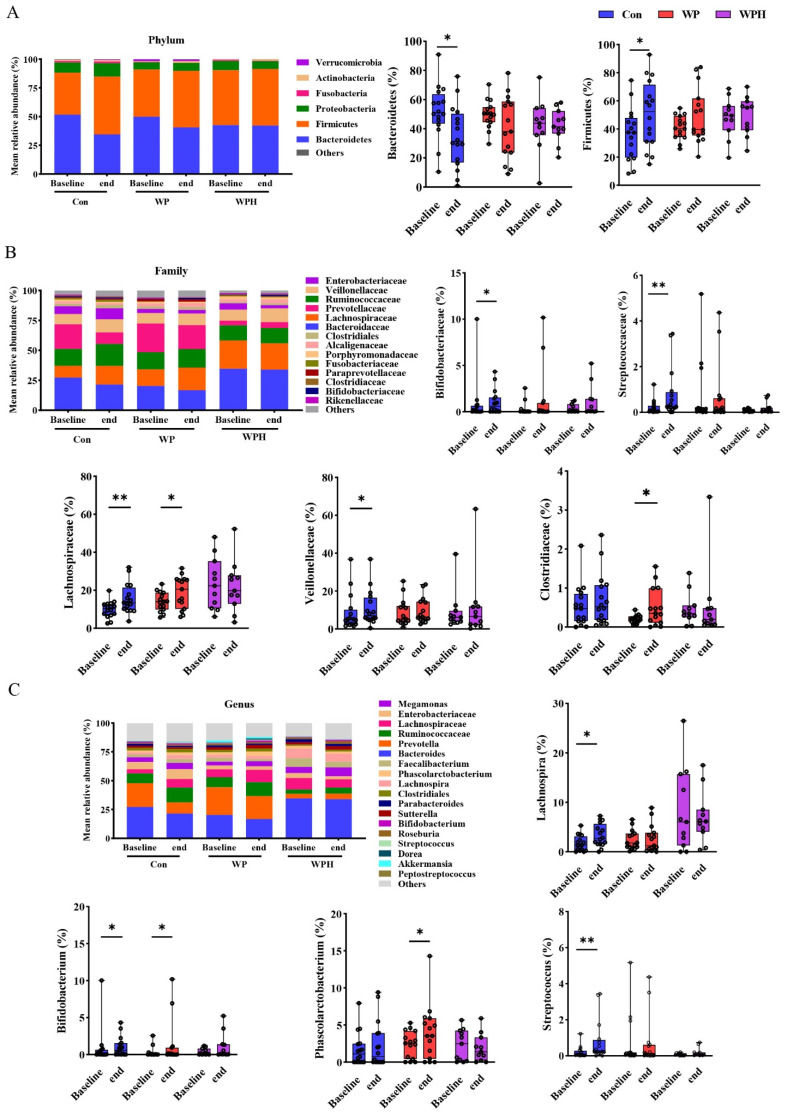
Changes in overall gut microbiota at three levels after intervention in each group, at the phylum (**A**), family (**B**), and genus (**C**) levels. * *p* < 0.05, ** *p* < 0.01.

In the α diversity analysis, no significant change was observed after intervention among the three groups based on Chao1, Ace, and Shannon (Figure 3). No significant changes in gut microbiota composition were observed at the phylum, the family, and the genus levels when compared among the three groups. At the phylum level, Bacteroidetes and Firmicutes were dominant in human intestinal bacteria. In the control group, Bacteroidetes were significantly decreased and Firmicutes were significantly increased compared with baseline. No significant differences were observed in the ratio of Bacteroidetes and Firmicutes (B/F) between baseline and end of intervention among the three groups (Figure 4A). At the family level, the percentage of Bifidobacteriaceae, Lachnospiraceae, Streptococcaceae, and Veillonellaceae was significantly increased compared with the baseline in the control group. WP intervention significantly increased the percentage of Lachnospiraceae and Clostridiaceae (Figure 4B). At the genus level, the percentage of Bifidobacterium, Lachnospira, and Streptococcus was significantly increased compared with baseline in the control group. WP intervention significantly increased the percentage of Bifidobacterium and Phascolarctobacterium (Figure 4C). Bacteria with significant differences among the three groups were not found via SEfSe analysis.

## 4. Discussion

Loss of skeletal muscle (sarcopenia) progresses with age and is a tough issue in the elderly with obesity. Caloric reduction combined with appropriate protein supplementation may prevent muscle loss. Therefore, we compared the effect of WP and WPH supplementation in an energy-restricted diet on body weight and muscle mass in older women with overweight and obesity. We found that an eight-week energy-restricted diet significantly decreased body weight and fat mass, with more noticeable results in the WPH group. Fat-free mass was preserved to a great extent in the WP and WPH groups (vs. the control group), but the change was not significant. Similar to our trial, Coker et al. [2] conducted an eight-week caloric restriction (lost 7% weight) in older adults with obesity. WP and essential amino acid (EAA) meals promoted a greater reduction in adipose tissue than control isocaloric meals. Although WP and EAA meals appeared to foster greater preservation of lean tissue, no significant difference was observed. Resistance exercise is beneficial for muscle maintenance and was combined with diet control in the weight loss studies. Oikawa et al. found that WP-isolates supplementation cannot alleviate the loss of muscle mass in older persons during physical inactivity and hypoenergetic states. However, WP isolate can offset muscle loss by augmenting muscle protein synthesis during recovery [19]. In healthy older women without hypoenergetic states, they found that WP isolates combined with resistance exercise resulted in further stimulation of skeletal muscle protein synthesis, reinforcing the importance of exercise in the maintenance of skeletal muscle health [3]. Verreijen et al. [20] also found that a high WP-, leucine-, and vitamin D-enriched supplement preserves appendicular muscle mass in obese older adults during a hypocaloric diet and resistance exercise program, potentially reducing the risk of sarcopenia. In the present trial, although resistance exercise was not prescribed, protein supplementation accelerated the decrease of weight and fat. Although this program did not increase muscle mass, protein supplementation maintained muscle mass. Considering the difficulty of resistance exercise, WP, especially WPH, is beneficial in older women with obesity during a weight-loss program.

In the present study, WPH, instead of WP, supplementation had a BP-lowering effect. On the one hand, the weight loss in the WPH group was greater than in the WP group, and the greater weight loss was accompanied by an improvement in blood pressure. On the other hand, It is reported that milk-derived peptides have a hypotensive effect via inhibition of the angiotensin-converting enzyme (ACE) [21]. 

For the metabolism of glucose and lipids, WP supplementation significantly decreased serum TG. Rakvaag et al. [22] reported that 60 g WPH for 12 weeks had beneficial effects on the postprandial TG area under the curve and fasting TG. In our previous meta-analysis of 13 trials, whey supplementation significantly reduced the circulating TG level, whereas the WP had no effects on circulating TC, LDL-C, and HDL-C [23]. Unexpectedly, WP increased fasting glucose and insulin levels, resulting in higher HOMA-IR. Several long-term interventions with WP isolate have been unable to detect effects on insulin sensitivity [24,25]. The acute insulinotropic potential of WP was observed in type 2 diabetic subjects [26]. High protein could have caused alterations in muscle cell structure and organization involved in the muscle insulin action [27]. In the present trial, we observed improved insulin resistance by WPH relative to WP supplementation. Thus, responsible mechanisms are based on the type and amount of WP products, the intervention method (hypocaloric diet, resistance exercise), and the health status of the subjects. Another point is that mean BP, glucose, lipid levels, and inflammatory response were within the normal range in these healthy older individuals. Hence, it could explain these changes in clinical significance, but the results should be taken with caution.

The tricarboxylic acid (TCA) cycle plays an important role in metabolism. The fluency of the TCA cycle is very important for maintaining normal cellular function, especially energy output [28]. A previous study reported that TCA cycling and pyruvate metabolism were downregulated in fat and skeletal muscle tissues in heavier subjects [29]. We found that WPH increased citric acid and pyruvic acid concentration, leading to the upregulation of the TCA cycle. Therefore, weight loss and fat reduction after WPH supplementation are involved in energy expenditure. However, direct evidence should be obtained in further trials. Circulating BCAAs significantly rise in people with obesity and are associated with diabetes risk [27]. We were concerned that BCAA-enriched WPH and WP will further increase BCAA in the blood, but it did not, which could be because the amount of protein intake was inadequate to alter the serum BCAA abundance. Similarly, during an eight-week weight-loss intervention, 20 g whey-based protein supplements did not affect postintervention plasma abundances of BCAA in obese women with metabolic syndrome [30].

The gut microbiome has been viewed as a predictor of health, and the environment and diet can influence the composition or function of the gut microbiome [11,31,32]. An energy-restricted high-protein diet benefits the microbiota by increasing intestinal Bifidobacterium and Lactobacillus [33]. However, very few reports can be found in the literature on the effect of whey protein on microbiota, particularly in humans. Reimer et al. [34] found that WP intervention for 12 weeks significantly reduced body fat without affecting the microbial community structure. Moreno-Pérez et al. [35] reported that athletes supplemented with WP isolate and beef hydrolysate had decreased beneficial bacteria, and increased the Bacteroides phylum belonging to malefic bacteria. A systematic review suggests that milk and yogurt consumption appeared to facilitate the growth of functional phylotypes related to improved host health. Only two trials using dairy derivatives (whey and casein isolates) revealed negligible effects on the gut microbiota [36]. In our study, WP supplementation induced somewhat taxonomic (family and genus) shifts compared to baseline. However, we did not find a change in microbial diversity after WP and WPH supplementation compared with the control. Interestingly, an animal study showed that gut microbiota composition and function changed after five weeks of intervention with a high-fat diet and WP isolate. However, such changes were no longer observed after 10 weeks of intervention, suggesting that the efficacy of this dietary intervention is age-dependent [37]. A further animal study showed that depletion of gut microbiota did not indicate the positive effects exerted by WP isolate in high-fat diet-induced obese mice, suggesting that WP might exploit other mechanisms, independent of gut microbiota, to protect the host from obesity [38]. In other words, gut microbiota in the obese state may obscure the effect of WP, especially in older subjects.

As far as we know, this study is the first trial conducted on the Chinese elder population to observe the whey protein effect in a weight-loss intervention. The protein intake of the elderly in China is lower than that in western countries. In our study, an energy-restricted diet was recommended according to the ideal body weight of individuals, which was more accurate than a consistent calorie recommendation. However, our study has some limitations: (1) the bitter taste of hydrolyzed protein makes double-blind design impossible; (2) although personalized dietary guidance was instructed by the dietitian, standard meals were not provided for participants; and (3) high interindividual variations in the gut microbiota may mask minor changes in distinct taxonomic diversity.

In conclusion, an energy-restricted diet reduced weight and fat mass in older women with overweight and obesity. WP and WPH supplementation further improved body composition. WPH was more effective in this improvement, and its mechanism is possibly associated with increased energy metabolism.

## Figures and Tables

**Table 1:** The baseline characteristics of study subjects who completed the study.

Characteristics	Total (*n* = 48)
Age (years)	61.33 ± 7.73
Body weight (kg)	65.66 ± 5.15
BMI (kg/m^2^)	27.21 ± 1.61
Energy intake (kcal)	1458.17 ± 301.23
Protein intake (g)	44.15 (36.53, 53.03)
Carbohydrate intake (g)	222.26 ± 52.20
Fat intake (g)	38.75 (33.78, 45.73)
PASE	112.98 (89.53, 133.18)

BMI, body mass index; PASE: physical activity scale for the elderly.

**Table 2:** The energy and macronutrient intake during the intervention.

	Control	WP	WPH
Energy-restricted diet			
Protein (g)	50.15 (45.99, 54.43)	53.30 (48.35, 56.34)	52.40 (50.38, 55.33)
Carbohydrate (g)	137.91 (126.47, 149.67)	146.58 (132.96, 154.93)	144.10 (138.53, 152.14)
Fat (g)	27.86 (25.55, 30.24)	29.61 (26.86, 31.30)	29.11 (27.99,30.74)
Energy (kcal)	1003.00 (919.75, 1088.50)	1066.00 (967.00, 1126.75)	1048.00 (1007.50, 1106.50)
Intervention			
Supplementary Protein (g)		15.2	16.8
Total protein intake (g)	50.15 (45.99, 54.43)	68.50 (63.55, 71.53) ^##^	69.24 (67.22, 72.17) ^##^
Protein (g)/weight (kg)	0.78 ± 0.05	1.01 ± 0.07 ^##^	1.08 ± 0.06 ^##^
Total energy intake (kcal)	1003.00 (919.75, 1088.50)	1126.80 (1027.80, 1187.55) ^##^	1115.36 (1074.86, 1173.86) ^##^

^##^ *p* < 0.01 vs. the control group.

**Table 3:** The changes in body composition among the three groups.

	Control			WP			WPH		
	Baseline	End	Change	Baseline	End	Change	Baseline	End	Change
Body weight (kg)	64.67 ± 4.00	63.57 ± 4.14 **	−1.11 ± 1.11	67.93 ± 4.58	66.44 ± 4.35 **	−1.49 ± 1.06	64.34 ± 6.41	62.00 ± 6.26 **	−2.34 ± 1.35 ^#^
BMI (kg/m^2^)	27.38 ± 1.37	26.92 ± 1.54 **	−0.46 ± 0.45	27.72 ± 1.71	27.13 ± 1.83 **	−0.59 ± 0.42	26.41 ± 1.57	25.44 ± 1.62 **	−0.97 ± 0.54 ^#^
Body fat (kg)	23.83 ± 2.92	23.13 ± 2.91 **	−0.70 ± 0.92	25.51 ± 2.78	24.04 ± 2.89 **	−1.46 ± 1.16	24.71 ± 3.49	22.26 ± 3.12 **	−2.45 ± 1.65 ^##^
Body fat (%)	36.45 (33.80, 39.70)	36.85 (35.00, 38.70)	−0.83 (−1.40, 0.60)	38.00 (34.53, 39.70)	36.50 (33.50, 37.75) **	−1.25 (−2.48, −0.72)	38.00 (35.93, 40.70)	35.70 (33.63, 38.83) **	−2.55 (−3.63, −0.80) ^#^
Visceral fat area (cm^2^)	113.36 ± 21.31	109.48 ± 22.42	−3.88 ± 8.72	121.82 ± 16.55	112.41 ± 18.73 **	−9.41 ± 8.32	122.52 ± 23.72	107.21 ± 22.78 **	−15.31 ± 10.11 ^##^
Fat-Free Mass (kg)	40.85 ± 3.14	40.44 ± 3.25	−0.41 ± 1.04	42.42 ± 3.61	42.39 ± 3.72	−0.03 ± 1.11	39.64 ± 4.28	39.74 ± 4.58	0.11 ± 0.84

** *p* < 0.01 vs. the baseline; ^#^ *p* < 0.05 vs. the changes in the control group, ^##^ *p* < 0.01 vs. the changes in the control group.

**Table 4:** The changes in biochemical blood markers among the three groups.

	Control			WP			WPH		
	Baseline	End	Change	Baseline	End	Change	Baseline	End	Change
SBP (mmHg)	133.00 (123.00, 151.00)	130.25 (125.80, 135.33)	1.00 (−11.00, 10.67)	133.50 (125.00, 147.50)	130.80 (127.50, 136.00)	−1.30 (−10.00, 2.50)	132.75 (129.00, 140.25)	122.67 (119.33, 127.50) **	−9.75 (−13.75, −6.00)
DBP (mmHg)	80.00 (76.00, 84.50)	79.60 (75.33, 85.00)	0.40 (−4.25, 4.25)	83.00 (75.00, 88.67)	85.20 (80.00, 87.33)	1.67 (−5.50, 9.33)	85.00 (79.60, 92.50)	80.17 (74.00, 81.50)	−3.00 (−16.50, 1.00)
FBG (mmol/L)	5.20 (4.99, 5.88)	5.34 (5.12, 5.98)	0.05 (−3.00, 4.33)	5.08 (4.82, 5.29)	5.13 (4.96, 5.56) *	0.20 (0.03, 0.38)	5.76 (5.59, 6.06)	5.66 (5.29, 6.03)	0.05 (−0.35, 0.26)
FINS (pmol/L)	55.65 (43.47, 88.15)	48.41 (35.54, 77.58)	−10.18 (−17.49, 11.07)	64.85 (51.07, 95.44)	83.26 (66.98, 104.90) *	10.60 (−7.21, 26.54)	74.39 (45.62, 98.56)	58.88 (44.44, 69.35)	−9.18 (−32.53, 9.32)
HOMA-IR	1.91 (1.41, 2.96)	1.52 (1.22, 2.65)	−0.39 (−0.64, 0.30)	2.05 (1.89, 3.15)	2.80 (2.21, 3.76) *	0.46 (0.03, 0.84)	2.75 (1.72, 3.58)	2.26 (1.51, 2.31)	−0.48 (−1.12, 0.33) ^&^
TC (mmol/L)	4.90 (4.32, 5.70)	4.82 (4.40, 5.63)	0.02 (−0.69, 0.55)	5.13 (4.61, 5.70)	5.05 (4.38, 5.62)	−0.14 (−0.49, 0.04)	5.43 (5.06, 6.13)	5.74 (5.37, 6.13)	0.26 (−0.37, 0.34)
TG (mmol/L)	1.57 (1.05, 2.85)	1.12 (0.72, 2.23)	−0.37 (−0.69, 0.04)	1.78 (1.39, 2.20)	1.62 (1.10, 1.88) **	−0.27 (−0.49, −0.11)	1.26 (1.05, 2.17)	1.50 (1.09, 1.77)	−0.07 (−0.59, 0.27)
LDL-C (mmol/L)	2.82 (2.33, 3.31)	2.83 (2.40, 3.41)	0.08 (−0.02, 0.26)	3.32 (2.58, 3.49)	3.17 (2.39, 3.71)	−0.08 (−0.43, 0.13)	3.61 (2.67, 4.02)	3.89 (2.94, 4.14)	0.18 (−0.25, 0.34)
HDL-C (mmol/L)	1.32 (1.13, 1.47)	1.29 (1.14, 1.59)	0.04 (−0.06, 0.11)	1.25 (1.06, 1.39)	1.30 (1.08, 1.38)	0.01 (−0.07, 0.09)	1.44 (1.20, 1.68)	1.43 (1.30, 1.85)	0.01 (−0.07, 0.23)
CRP (mg/L)	0.86 (0.56, 1.76)	0.91 (0.53, 1.66)	0.01 (−0.44, 0.27)	1.36 (0.82, 2.17)	1.48 (1.15, 2.66)	0.11 (−0.32, 0.72)	1.69 (0.74, 7.11)	1.34 (0.69, 2.28)	−0.24 (−3.64, 0.15)
TNF-α (pg/mL)	100.50 (87.84, 137.86)	101.68 (88.61, 113.32)	0.43 (−25.56, 18.58)	86.93 (74.98, 99.35)	84.62 (74.81, 105.72)	0.59 (−11.78, 19.26)	92.29 (81.41, 103.16)	101.51 (87.54, 123.85)	20.71 (−6.19, 31.57)
FFA (μg/mL)	17.68 (12.54, 21.46)	16.90 (12.03, 22.11)	−0.40 (−1.48, 1.24)	14.88 (10.50, 21.44)	15.03 (11.55, 23.50)	0.47 (−2.07, 2.71)	12.47 (9.73, 14.43)	13.54 (11.10, 16.48)	1.42 (−0.004, 3.87)
UA (μmol/L)	267.05 (233.75, 348.63)	274.75 (222.50, 330.45)	3.60 (−32.25, 32.65)	300.00 (275.00, 330.23)	303.00 (249.80, 327.30)	2.15 (−32.53, 23.00)	316.75 (280.93, 354.70)	301.60 (276.43, 334.90)	−16.45 (−49.83, 17.40)

SBP, systolic blood pressure; DBP, diastolic blood pressure; FBG, fasting blood sugar; HOMA-IR, homeostatic model assessment of insulin resistance; UA, uric acid; TC, total cholesterol; TG, triglyceride; * *p* < 0.05, ** *p* < 0.01, compared with baseline; ^&^ *p* < 0.05, compared with the changes of WP group.

## Data Availability

The datasets used or analyzed during the current study are available from the corresponding author on reasonable request.

## Supplementary Materials

The following supporting information can be downloaded at: https://www.mdpi.com/article/10.3390/nu14214540/s1, Table S1: the components of 100 g of supplements; Table S2: pathways significantly enriched for metabolites associated with change between treatment groups.

## References

[1] Pan X.-F., Wang L., Pan A. (2021). Epidemiology and determinants of obesity in China. Lancet Diabetes Endocrinol..

[2] Coker R.H., Miller S., Schutzler S., Deutz N., Wolfe R.R. (2012). Whey protein and essential amino acids promote the reduction of adipose tissue and increased muscle protein synthesis during caloric restriction-induced weight loss in elderly, obese individuals. Nutr. J..

[3] Oikawa S.Y., Kamal M.J., Webb E.K., McGlory C., Baker S.K., Phillips S.M. (2020). Whey protein but not collagen peptides stimulate acute and longer-term muscle protein synthesis with and without resistance exercise in healthy older women: A randomized controlled trial. Am. J. Clin. Nutr..

[4] Longland T.M., Oikawa S.Y., Mitchell C.J., Devries M.C., Phillips S.M. (2016). Higher compared with lower dietary protein during an energy deficit combined with intense exercise promotes greater lean mass gain and fat mass loss: A randomized trial. Am. J. Clin. Nutr..

[5] Wright C.S., Zhou J., Sayer R.D., Kim J.E., Campbell W.W. (2018). Effects of a High-Protein Diet Including Whole Eggs on Muscle Composition and Indices of Cardiometabolic Health and Systemic Inflammation in Older Adults with Overweight or Obesity: A Randomized Controlled Trial. Nutrients.

[6] Kim J.E., O’Connor L.E., Sands L.P., Slebodnik M.B., Campbell W.W. (2016). Effects of dietary protein intake on body composition changes after weight loss in older adults: A systematic review and meta-analysis. Nutr. Rev..

[7] Sammarco R., Marra M., Di Guglielmo M.L., Naccarato M., Contaldo F., Poggiogalle E., Donini L.M., Pasanisi F. (2017). Evaluation of Hypocaloric Diet with Protein Supplementation in Middle-Aged Sarcopenic Obese Women: A Pilot Study. Obes. Facts.

[8] Putra C., Konow N., Gage M., York C.G., Mangano K.M. (2021). Protein Source and Muscle Health in Older Adults: A Literature Review. Nutrients.

[9] Lee J. (2014). Nutrition: A Survey on Intake of Protein Supplement of University Students Majoring in Physical Education. J. Korean Soc. Food Sci. Nutr..

[10] Cox A.J., West N.P., Cripps A.W. (2015). Obesity, inflammation, and the gut microbiota. Lancet Diabetes Endocrinol..

[11] Rothschild D., Weissbrod O., Barkan E., Kurilshikov A., Korem T., Zeevi D., Costea P.I., Godneva A., Kalka I.N., Bar N. (2018). Environment dominates over host genetics in shaping human gut microbiota. Nature.

[12] Beaumont M., Portune K.J., Steuer N., Lan A., Cerrudo V., Audebert M., Dumont F., Mancano G., Khodorova N., Andriamihaja M. (2017). Quantity and source of dietary protein influence metabolite production by gut microbiota and rectal mucosa gene expression: A randomized, parallel, double-blind trial in overweight humans. Am. J. Clin. Nutr..

[13] Geidenstam N., Al-Majdoub M., Ekman M., Spegel P., Ridderstrale M. (2017). Metabolite profiling of obese individuals before and after a one year weight loss program. Int. J. Obes..

[14] Stroeve J.H., Saccenti E., Bouwman J., Dane A., Strassburg K., Vervoort J., Hankemeier T., Astrup A., Smilde A.K., van Ommen B. (2016). Weight loss predictability by plasma metabolic signatures in adults with obesity and morbid obesity of the DiOGenes study. Obesity.

[15] Pedersen H.K., Gudmundsdottir V., Nielsen H.B., Hyotylainen T., Nielsen T., Jensen B.A., Forslund K., Hildebrand F., Prifti E., Falony G. (2016). Human gut microbes impact host serum metabolome and insulin sensitivity. Nature.

[16] Chen W. (2021). Chinese Guidelines for Medical Nutrition treatment of Overweight/Obesity (2021). Asia Pac. J. Clin. Nutr..

[17] Washburn R.A., Smith K.W., Jette A.M., Janney C.A. (1993). The Physical Activity Scale for the Elderly (PASE): Development and evaluation. J. Clin. Epidemiol..

[18] Wan C., Zhu C., Jin G., Zhu M., Hua J., He Y. (2021). Analysis of gut microbiota in patients with coronary artery disease and hypertension. Evid.-Based Complementary Altern. Med..

[19] Oikawa S.Y., McGlory C., D’Souza L.K., Morgan A.K., Saddler N.I., Baker S.K., Parise G., Phillips S.M. (2018). A randomized controlled trial of the impact of protein supplementation on leg lean mass and integrated muscle protein synthesis during inactivity and energy restriction in older persons. Am. J. Clin. Nutr..

[20] Verreijen A.M., Verlaan S., Engberink M.F., Swinkels S., de Vogel-van den Bosch J., Weijs P.J. (2015). A high whey protein-, leucine-, and vitamin D-enriched supplement preserves muscle mass during intentional weight loss in obese older adults: A double-blind randomized controlled trial. Am. J. Clin. Nutr..

[21] Xu J.Y., Qin L.Q., Wang P.Y., Li W., Chang C. (2008). Effect of milk tripeptides on blood pressure: A meta-analysis of randomized controlled trials. Nutrition.

[22] Rakvaag E., Fuglsang-Nielsen R., Bach Knudsen K.E., Landberg R., Johannesson Hjelholt A., Sondergaard E., Hermansen K., Gregersen S. (2019). Whey Protein Combined with Low Dietary Fiber Improves Lipid Profile in Subjects with Abdominal Obesity: A Randomized, Controlled Trial. Nutrients.

[23] Zhang J.W., Tong X., Wan Z., Wang Y., Qin L.Q., Szeto I.M. (2016). Effect of whey protein on blood lipid profiles: A meta-analysis of randomized controlled trials. Eur. J. Clin. Nutr..

[24] Fuglsang-Nielsen R., Rakvaag E., Langdahl B., Knudsen K.E.B., Hartmann B., Holst J.J., Hermansen K., Gregersen S. (2021). Effects of whey protein and dietary fiber intake on insulin sensitivity, body composition, energy expenditure, blood pressure, and appetite in subjects with abdominal obesity. Eur. J. Clin. Nutr..

[25] Fekete A.A., Giromini C., Chatzidiakou Y., Givens D.I., Lovegrove J.A. (2016). Whey protein lowers blood pressure and improves endothelial function and lipid biomarkers in adults with prehypertension and mild hypertension: Results from the chronic Whey2Go randomized controlled trial. Am. J. Clin. Nutr..

[26] Frid A.H., Nilsson M., Holst J.J., Björck I.M.E. (2005). Effect of whey on blood glucose and insulin responses to composite breakfast and lunch meals in type 2 diabetic subjects. Am. J. Clin. Nutr..

[27] Zheng Y., Ceglarek U., Huang T., Li L., Rood J., Ryan D.H., Bray G.A., Sacks F.M., Schwarzfuchs D., Thiery J. (2016). Weight-loss diets and 2-y changes in circulating amino acids in 2 randomized intervention trials. Am. J. Clin. Nutr..

[28] Wang Z., Zhang F., Liu W., Sheng N., Sun H., Zhang J. (2021). Impaired tricarboxylic acid cycle flux and mitochondrial aerobic respiration during isoproterenol induced myocardial ischemia is rescued by bilobalide. J. Pharm. Anal..

[29] van der Kolk B.W., Saari S., Lovric A., Arif M., Alvarez M., Ko A., Miao Z., Sahebekhtiari N., Muniandy M., Heinonen S. (2021). Molecular pathways behind acquired obesity: Adipose tissue and skeletal muscle multiomics in monozygotic twin pairs discordant for BMI. Cell Rep. Med..

[30] Piccolo B.D., Comerford K.B., Karakas S.E., Knotts T.A., Fiehn O., Adams S.H. (2015). Whey protein supplementation does not alter plasma branched-chained amino acid profiles but results in unique metabolomics patterns in obese women enrolled in an 8-week weight loss trial. J. Nutr..

[31] Valdes A.M., Walter J., Segal E., Spector T.D. (2018). Role of the gut microbiota in nutrition and health. BMJ.

[32] Jackson M.A., Verdi S., Maxan M.E., Shin C.M., Zierer J., Bowyer R.C.E., Martin T., Williams F.M.K., Menni C., Bell J.T. (2018). Gut microbiota associations with common diseases and prescription medications in a population-based cohort. Nat. Commun..

[33] Dong T.S., Luu K., Lagishetty V., Sedighian F., Woo S.L., Dreskin B.W., Katzka W., Chang C., Zhou Y., Arias-Jayo N. (2020). A High Protein Calorie Restriction Diet Alters the Gut Microbiome in Obesity. Nutrients.

[34] Reimer R.A., Willis H.J., Tunnicliffe J.M., Park H., Madsen K.L., Soto-Vaca A. (2017). Inulin-type fructans and whey protein both modulate appetite but only fructans alter gut microbiota in adults with overweight/obesity: A randomized controlled trial. Mol. Nutr. Food Res..

[35] (2018). Moreno-Perez D, Bressa C, Bailen M, Hamed-Bousdar S, Naclerio F, Carmona M, Perez M, Gonzalez-Soltero R, Montalvo-Lominchar MG, Carabana C, Larrosa M: Effect of a Protein Supplement on the Gut Microbiota of Endurance Athletes: A Randomized, Controlled, Double-Blind Pilot Study. Nutrients.

[36] Aslam H., Marx W., Rocks T., Loughman A., Chandrasekaran V., Ruusunen A., Dawson S.L., West M., Mullarkey E., Pasco J.A. (2020). The effects of dairy and dairy derivatives on the gut microbiota: A systematic literature review. Gut Microbes.

[37] Boscaini S., Cabrera-Rubio R., Nychyk O., Roger Speakman J., Francis Cryan J., David Cotter P., Nilaweera K.N. (2020). Age- and duration-dependent effects of whey protein on high-fat diet-induced changes in body weight, lipid metabolism, and gut microbiota in mice. Physiol. Rep..

[38] Boscaini S., Cabrera-Rubio R., Golubeva A., Nychyk O., Fulling C., Speakman J.R., Cotter P.D., Cryan J.F., Nilaweera K.N. (2021). Depletion of the gut microbiota differentially affects the impact of whey protein on high-fat diet-induced obesity and intestinal permeability. Physiol. Rep..

