# Supplementary material for: Effects of Whey Protein or Its Hydrolysate Supplements Combined with an Energy-Restricted Diet on Weight Loss: A Randomized Controlled Trial in Older Women
Source: Nutrients. 2022 Oct 28;14(21):4540. doi: 10.3390/nu14214540 (PMC9657015; doi:10.3390/nu14214540)
Supplement: Supplementary file 1 [file nutrients-14-04540-s001.zip › Table S1.pdf]

Supplementary Table S1. The components for each package (10g)of supplements

	WP	WPH
Protein (g)	7.6	8.4
Carbohydrate (g)	0.46	0.20
Fat (g)	0.77	0.01
Saturated fat (g)	0.49	0.01
Trans fat (g)	0.03	0.01
Cholesterol (g)	0.015	0
Energy (kcal)	165.5	146.9
