# Supplementary material for: Effects of Whey Protein or Its Hydrolysate Supplements Combined with an Energy-Restricted Diet on Weight Loss: A Randomized Controlled Trial in Older Women
Source: Nutrients. 2022 Oct 28;14(21):4540. doi: 10.3390/nu14214540 (PMC9657015; doi:10.3390/nu14214540)
Supplement: Supplementary file 1 [file nutrients-14-04540-s001.zip › Table S2.pdf]

Supplementary Table S2. Pathways significantly enriched for metabolites associated with change between treatment groups

	Metabolites	VIP	trend	t.stat	p.value	FDR	Pathway Name
Comparison among the group changes							
WP-control	Mevalonic acid	2.04	↑**	3.31	0.002	0.046	Terpenoid backbone biosynthesis
WPH-control	Citric acid	1.89	↑**	4.20	<0.001	0.005	Citrate cycle (TCA cycle); Glyoxylate and dicarboxylate metabolism
	Pyruvic acid	1.68	↑**	3.42	0.002	0.020	Pyruvate metabolism;Tyrosine metabolism; TCA cycle; Glyoxylate and dicarboxylate metabolism; Glycolysis/ Gluconeogenesis
	Glyceric acid	1.64	↑**	3.41	0.002	0.020	Glyoxylate and dicarboxylate metabolism; Glycerolipid metabolism
WPH-WP	Citric acid	1.86	↑**	3.22	0.004	0.047	TCA cycle
Comparison between end and baseline							
Control	Tyrosine	2.36	↑**	5.44	<0.001	<0.001	Phenylalanine, tyrosine and tryptophan biosynthesis; Tyrosine metabolism
	PC(18:4(6Z,9Z,12Z,15Z)/P-18:1(11Z))	1.90	↑**	4.11	<0.001	0.004	Arachidonic acid metabolism; Glycerophospholipid metabolism
	Leukotriene C4	1.77	↓**	-3.45	0.002	0.014	Arachidonic acid metabolism

	Methionine	1.70	↑**	3.15	0.003	0.029	Cysteine and methionine metabolism
WP	gamma-Aminobutyric acid	2.05	↑**	3.54	0.001	0.032	Alanine, aspartate and glutamate metabolism
	Tryptophan	1.97	↑**	3.39	0.002	0.037	Tryptophan metabolism
WPH	gamma-Aminobutyric acid	2.02	↑**	4.03	0.001	0.013	Alanine, aspartate and glutamate metabolism
	2-Oxobutyric acid	1.93	↑**	3.45	0.003	0.037	Cysteine and methionine metabolism
	Histidine	1.89	↓**	-3.45	0.003	0.037	Histidine metabolism

**P<0.01. FDR, false discovery rate.
